# The Effect of Orally Dosed Levagen+™ (palmitoylethanolamide) on Exercise Recovery in Healthy Males—A Double-Blind, Randomized, Placebo-Controlled Study

**DOI:** 10.3390/nu12030596

**Published:** 2020-02-25

**Authors:** Alistair Mallard, David Briskey, Andrew Richards, Dean Mills, Amanda Rao

**Affiliations:** 1 RDC Clinical, Brisbane 4006, QLD, Australia; 2 School of Human Movement and Nutrition Sciences, The University of Queensland, Brisbane 4067, QLD, Australia; 3 Respiratory and Exercise Physiology Research Group, School of Health and Wellbeing, University of Southern Queensland, Ipswich 4305, QLD, Australia; 4 Centre for Health, Informatics, and Economic Research, Institute for Resilient Regions, University of Southern Queensland, Ipswich 4305, QLD, Australia; 5 School of Medicine, University of Sydney, Sydney 2006, NSW, Australia

**Keywords:** palmitoylethanolamide, recovery, leg press exercise, muscle damage, functional foods

## Abstract

The aim of this study was to evaluate the effect of palmitoylethanolamide (PEA), a cannabimimetic compound and lipid messenger, on recovery from muscle damaging exercise. Twenty-eight healthy young male participants attended the laboratory four times on subsequent days. In the first visit, baseline characteristics were recorded before participants were randomized to consume either liquid PEA (167.5 mg Levagen+ with 832.5 mg maltodextrin) or a matched placebo (1 g maltodextrin) drink. Leg press exercise consisted of four sets at 80% of one repetition maximum followed by a performance set. Muscle soreness, thigh circumference, blood lactate concentration, biomarkers of muscle damage and inflammation, and transcription factor pathways were measured pre- and immediately post-exercise and again at 1, 2, 3, 24, 48, and 72 h post-exercise. The leg press exercise increased (*p* < 0.05) blood lactate concentration and induced muscle damage as evidenced by increased muscle soreness, thigh circumference, biomarkers of muscle damage, and concentrations of tumor necrosis factor-α. PEA reduced (*p* < 0.05) myoglobin and blood lactate concentrations and increased protein kinase B phosphorylation following exercise. Taken together, these results indicate PEA supplementation may aid in muscle recovery from repeat bouts of exercise performed within a short duration by reducing myoglobin and lactate concentration.

## 1. Introduction

Exercise-induced muscle damage is a phenomenon caused by unaccustomed exercise that is characterized by transient ultrastructural myofibrillar disruption [1,2]. Following exercise, there is muscle soreness, decreased pressure pain threshold, localized swelling, and temporary reductions in muscle strength, power, and range of motion in the affected limb [3]. The myofibrillar disruption is accompanied by the systemic efflux of myocellular enzymes and proteins, including creatine kinase, lactate dehydrogenase, and myoglobin. Exercise-induced muscle damage stimulates various cell types within skeletal muscle to initiate subsequent tissue repair and remodeling including satellite, inflammatory, vascular, and stromal cells that interact with each other within the extracellular matrix [1,2].

Exercise-induced muscle damage can limit recovery from a previous bout of exercise. Successful recovery would enable an individual to return to training and competition quicker, and possibly allow for higher exercise intensity to be performed [4]. Increasing the intensity and volume of training sessions in a given period may allow for improved performance in subsequent competitions [4]. Non-steroidal anti-inflammatory drugs are commonly prescribed to alleviate the symptoms of exercise-induced muscle damage. However, there is evidence to suggest that long-term use of such drugs may impair the skeletal muscle adaptive response to exercise and there are several reported side effects including stomach issues [5]. Thus, there is a direct need for sustainable, long-term, treatments with fewer potential side effects for the prevention and management of exercise-induced muscle damage [6].

Palmitoylethanolamide (PEA) is a cannabimimetic compound and lipid messenger found in a wide variety of food sources that is hypothesized to reduce pain through endocannabinoid driven activities or by reducing inflammation [7,8]. Several human studies investigated PEA as a treatment for pain and a recent systematic review and meta-analysis, that identified 10 studies including data from 786 participants who received PEA and 512 controls, demonstrated that PEA supplementation was associated with significantly greater pain reduction compared to inactive control conditions [9]. The mechanisms responsible for the reduction in pain may be due to PEA agonism of peroxisome proliferator activated receptor-α, which has been shown to have a major role in PEA mechanisms for pain relief [10]. PEA also has an essential role in the suppression of inflammation by reducing the activity of pro-inflammatory enzymes cyclooxygenase, endothelial nitric oxide synthase and inducible nitric oxide synthases [11], and mast cell activation [12,13]. These mechanisms of action may also reduce painful symptoms that result from exercise-induced muscle damage and would be advantageous for individuals who require rapid recovery between acute successive bouts of exercise (e.g., sports tournament) [14]. However, to our knowledge no research has undertaken this.

Accordingly, the aim of this study is to evaluate the effects of PEA on recovery from muscle-damaging exercise. We hypothesize that PEA supplementation immediately before and after exercise will reduce pain and decrease localized swelling through a reduction in pro-inflammatory intramuscular enzymes and cytokines. Additionally, based on the mechanisms of action of PEA on inflammation we hypothesize that PEA will also reduce muscle damage because of the inflammatory processes involved in muscle injury and repair.

## 2. Materials and Methods

### 2.1. Participants

Twenty-eight healthy young male participants with prior experience in resistance exercise training participated in the study and provided written informed consent prior to testing (Table 1). The exclusion criteria included any unstable or serious illness, use of long-term medications, malignancy or treatment for malignancy within the previous two years, tobacco use, medically prescribed diet, slimming diet, vegan diet, macrobiotic diet, chronic past or current alcohol abuse, allergy to any study ingredients, serious mood disorders, insomnia, night-shift employment, any diagnosed neurological conditions, recent musculoskeletal injuries, and participation in any other clinical trial in the past three months. The inclusion criteria included normal dietary habits, body mass index between 18.5 to 35 kg/m^2^, recreationally trained and achieving at least 150 min of exercise per week, and at least six months of resistance training experience in the past year. Experimental procedures were approved by the Bellberry Human Research Ethics Committee (HREC 2017-11-841), conformed to the Declaration of Helsinki and registered with the Australia and New Zealand Clinical Trials Registry (ANZCTRN1261800285257).

### 2.2. Experimental Design

**Figure 1:**
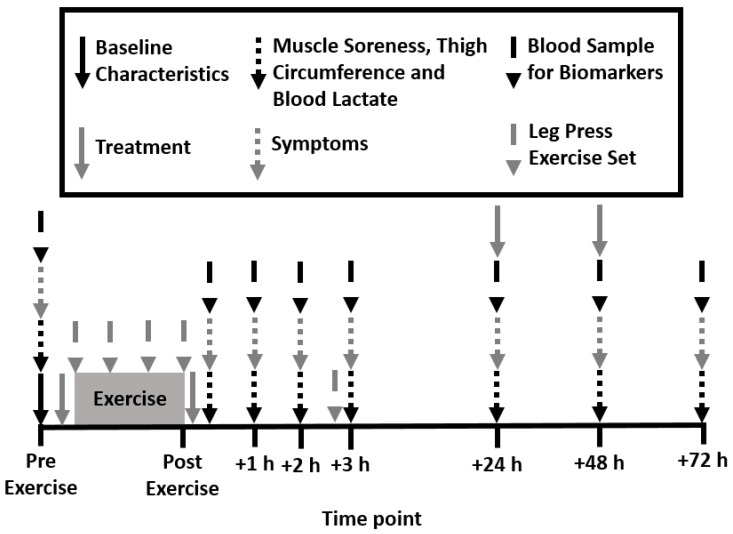
Schematic of experimental design.

A randomized, double-blind, placebo-controlled design was employed for this study. Participants attended the laboratory four times on subsequent days between 6–8 am (Figure 1). During the first visit, height, body mass, waist, hip and thigh circumference, resting brachial blood pressure, and heart rate (HEM-7121, Omron Healthcare Co., Kyoto, Japan) were measured. Participants were then familiarized with all testing procedures and randomized to consume either liquid PEA or a matched placebo drink pre- and post-exercise. Exercise consisted of four leg press sets at 80% of one repetition maximum (1-RM) and one performance set at 70% 1-RM. Muscle soreness, thigh circumference, blood lactate concentration, biomarkers of muscle damage and inflammation, and transcription factor pathways were measured pre- and immediately post-exercise and then again at 1, 2, 3, 24, 48, and 72 h post-exercise.

### 2.3. Treatments

The PEA group consumed 167.5 mg of Levagen+ (150 mg of PEA and 17.5 mg of excipients) with 832.5 mg of maltodextrin and the placebo group consumed 1 g of maltodextrin. Levagen+ contains palmitoylethanolamide, coconut oil (fractionated), polyglycerol polyricinoleate, citrus oil, olive oil, lecithin, dl-alpha tocopheryl acetate, and silicon dioxide. Previous research showed a two-fold increase in plasma concentrations of PEA after oral administration of 300 mg [15]. Each treatment was mixed with 250 mL of water in an opaque bottle. To adhere to the double-blind protocol, a third party not associated with the study created the pre-mixed drinks. Each treatment was provided at the following time points: pre-exercise, post-exercise, 24 h post-exercise, and 48 h post-exercise (Figure 1).

### 2.4. Determination of One Repetition Maximum Load

Thirty minutes after the consumption of the treatments, participants completed a 5 min warm-up at a self-selected pace on a cycle ergometer (Ergomedic 828 E, Monark, Vansbro, Sweden). Participants then stretched major muscle groups of the lower limbs followed by 10 repetitions of a leg press exercise (45 Degree Leg Press, Force USA, Draper, UT, USA) at 50% of estimated 1-RM followed by 2 min rest. The weight on the leg press was then increased to approximately 70% of the estimated 1-RM and participants completed 4 to 6 repetitions followed by 2 min rest. Weight was added onto the leg press to approximately 90% of estimated 1-RM and participants completed one repetition followed by 2 min rest. Weight was then increased to 100% of estimated 1-RM and participants attempted to complete a repetition. Participants were given up to 7 attempts to achieve 1-RM and were allowed to rest for 3 min in-between attempts. Once 1-RM testing was complete participants rested for 5 min.

### 2.5. Leg Press Exercise

Participants completed four sets of a leg press exercise at 80% of 1-RM. For each set, the participant was asked to perform as many repetitions as possible, until volitional exhaustion. Each repetition was completed as fast as possible with maximal intent. The participants rested for 60 s between each set. Each repetition was recorded using an accelerometer-based system attached to the leg press sled to determine repetition velocity and power (Push Band 2.0, Push Inc, Toronto, Canada). Once the fourth set was complete, participants rested for 5 min and were then taken through testing for their 1-RM again. At 2 h and 45 min after the second 1-RM test the participants performed a third 1-RM test after a 5 min self-selected pace cycling warm-up. After a 5 min rest period, the participants performed one set of leg press exercises at 70% of 1-RM achieved at baseline. The participants were instructed to complete as many repetitions as possible with maximal leg speed. A 5 min cycling warm-down was then completed at a self-selected pace.

### 2.6. Muscle Soreness, Thigh Circumference, and Blood Lactate Concentration

Muscle soreness was measured using a visual analog scale which consisted of a 10 cm line with end points labeled “no pain” (left) and “unbearable pain” (right). Thigh circumference was measured at the point equidistant from trochanterion and tibiale laterale in a relaxed standing position. A fingertip capillary blood sample was used to measure blood lactate concentration (The Edge Lactate Analyzer, Apex Biotechnology Corporation, Hsinchu City, Taiwan).

### 2.7. Biomarkers of Muscle Damage and Inflammation and Phosphoprotein Signaling Pathways

Blood was collected into ethylenediaminetetraacetic acid plasma and serum vacutainers (BD, Plymouth, UK). Plasma samples were immediately centrifuged, whilst serum samples were incubated at room temperature for 30 min before centrifugation at 1500× *g* for 10 min at 4 °C. Separated plasma and serum were aliquoted and stored at −80 °C until biochemical assays were performed. Serum creatine kinase, lactate dehydrogenase, myoglobin, and high sensitivity C-reactive protein were analyzed in duplicate using a clinical chemistry analyzer (BK400, Biobase, Jinan, China).

Mononuclear cells were collected using cell preparation tubes (CPT) (BD, Plymouth, UK). Briefly, whole blood collected in the CPTs was immediately centrifuged at 1500× *g* for 20 min at room temperature. Half of the separated plasma was aspirated and discarded whilst the remaining plasma and cell layer were transferred to a 15 mL conical centrifuge tube using a Pasteur pipette. Phosphate buffered saline (PBS) was added to the conical tube and the tube was inverted 5 times to wash the cell layer. The cell/PBS mix was centrifuged at 300 relative centrifugal force (RCF) for 15 min at room temperature. After removing the supernatant, the washing step was repeated with 10 mL of PBS. The remaining cell layer was resuspended after aspiration in PBS with a protease inhibitor cocktail (Sigma-Aldrich, Milwaukee, WI, USA) and stored at −80 °C until biochemical assays were performed.

Mononuclear cells were analyzed for protein kinase B, cAMP response element-binding protein, extracellular signal-regulated kinases 1/2, c-Jun N-terminal kinases, nuclear factor kappa-light-chain-enhancer of activated B cells, p38 mitogen-activated protein kinases, ribosomal protein S6 kinase beta-1, and signal transducer and activator of transcription 3 and 5 using a multi-pathway 9-plex magnetic bead kit (cat. # 48-680MAG, Merck, Darmstadt, Germany). Each well had a known amount of cell protein added to ensure all results were relative. Serum interleukin-10 and 6, and tumor necrosis factor-α were analyzed in duplicate (Luminex 200, Austin, TX, USA) with high sensitivity Milliplex kits and calibrators (Merck Millipore, Darmstadt, Germany).

### 2.8. Data Analyses

Statistical analyses were performed using IBM SPSS Statistics (version 25.0) for Windows (IBM, Chicago, IL, USA). A sample size of 14 per group was calculated based on the power to detect a change of 20% in creatine kinase (300 IU/L down to 240 IU/L); effect size: 0.857, alpha error prob: 0.05, power 0.8. This allowed for a 20% drop out. All data were confirmed as parametric via a Shapiro–Wilk test for normality. Baseline participant characteristics between groups were analyzed with an independent samples *t*-test. A two-way ANOVA was used to analyze the effects of “time” (sets 1–4 and performance set for exercise and pre-, immediately post-exercise, and 1, 2, 3, 24, 48, and 72 h post-exercise for all other measures) and “treatment” (PEA vs. placebo) with a Greenhouse–Geisser correction where appropriate. Where significant treatment baseline differences were apparent (interleukin-6, cAMP response element-binding protein), results were normalized to baseline values prior to subsequent statistical analysis. Significant between-treatment differences were further explored using one-way repeated-measures ANOVA. Planned pairwise comparisons were made with repeated measures *t*-tests and the Bonferroni post-hoc adjustment. Statistical significance was set at *p* < 0.05. Data are presented as mean ± SD.

## 3. Results

There were no between-group differences in baseline participant characteristics (Table 1), indicating that the groups were well matched prior to testing. No adverse effects of the treatments were reported by the participants throughout the trial.

### 3.1. Leg Press Exercise

**Figure 2:**
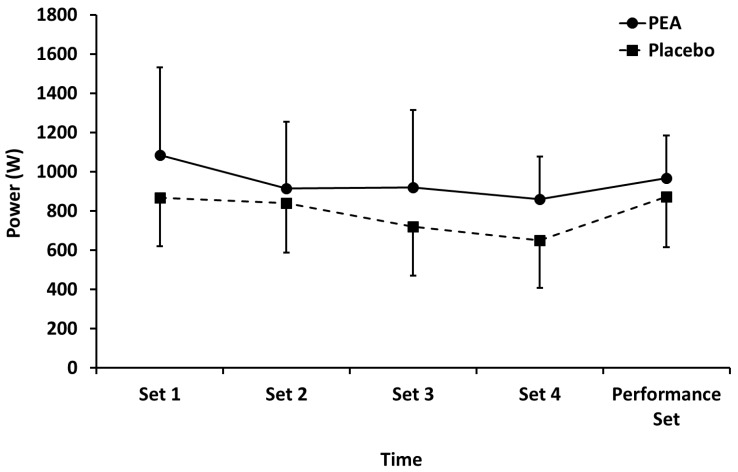
Mean power output during leg press exercise for palmitoylethanolamide (PEA) and placebo groups. Values are mean ± SD.

Mean power (Figure 2) performed during leg press exercise in both PEA and placebo groups decreased (*p* < 0.01) during sets 1–4, but there were no main effects of treatment or time by treatment interactions. There were also no between-group differences for the performance set.

### 3.2. Muscle Soreness, Thigh Circumference, and Blood Lactate Concentration

**Figure 3:**
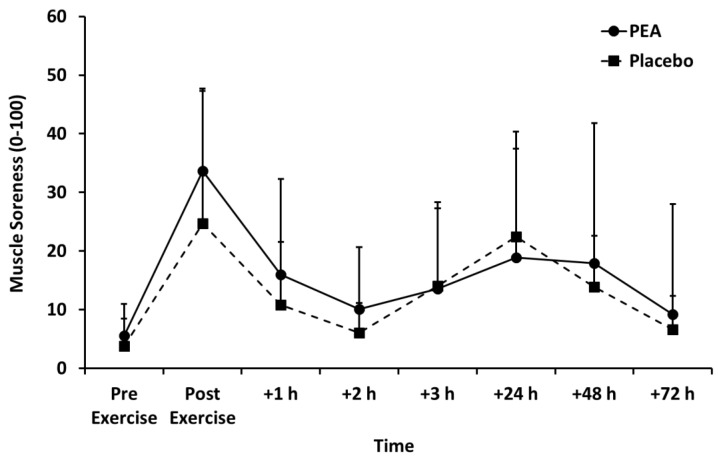
Muscle soreness for palmitoylethanolamide (PEA) and placebo groups. Values are mean ± SD.

**Figure 4:**
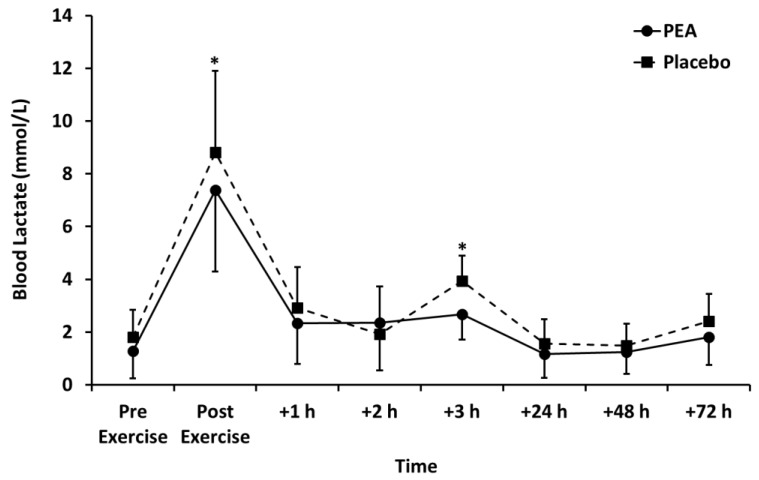
Blood lactate concentration for palmitoylethanolamide (PEA) and placebo groups. Values are mean ± SD. * *p* < 0.05 between groups.

Muscle soreness (Figure 3), thigh circumference (Table 2), and blood lactate concentration (Figure 4) peaked after set 2 of leg press exercise and declined thereafter (*p* < 0.01). Blood lactate concentration was lower for the PEA than placebo group at +1 and +3 h post-exercise. There were no main effects of treatment or time by treatment interactions for muscle soreness or thigh circumference.

### 3.3. Biomarkers of Muscle Damage

**Figure 5:**
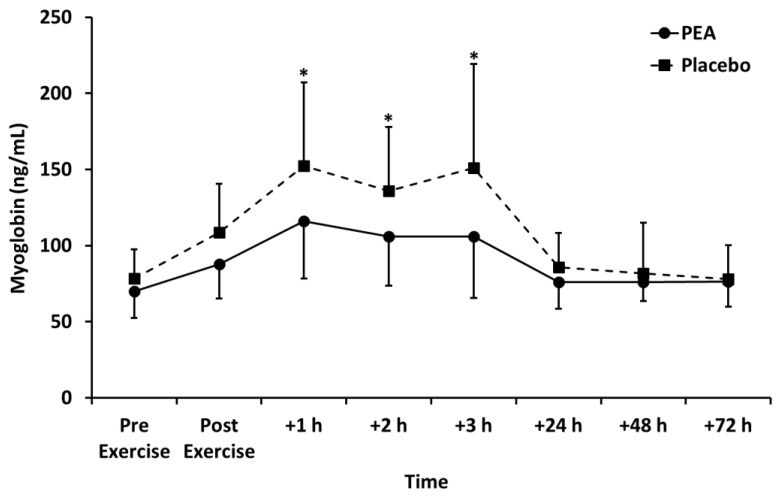
Myoglobin concentration for palmitoylethanolamide (PEA) and placebo groups. Values are mean ± SD. * *p* < 0.05 between groups.

Myoglobin concentration peaked +1 h after exercise and declined thereafter (*p* < 0.01; Figure 5). Myoglobin concentration was lower for the PEA than placebo group at +1, +2, and +3 h post exercise (*p* < 0.05). Creatine kinase peaked +24 h after exercise and decreased thereafter (*p* < 0.05). There was no change in lactate dehydrogenase over time. There were also no main effects of treatment or time by treatment interactions for creatine kinase and lactate dehydrogenase (Table 3).

### 3.4. Biomarkers of Inflammation

There were no main effects of time or treatment for interleukin-6 and 10 (Table 3), demonstrating that these inflammatory markers were not changed by the exercise. Tumor necrosis factor-α peaked post exercise and declined thereafter (*p* < 0.05), but there were no main effects of treatment or time by treatment interactions (Table 3).

### 3.5. Phosphoprotein Signaling Pathways

Protein kinase B phosphorylation peaked immediately after exercise for the PEA group only (*p* < 0.05; Table 2) and returned to baseline values 1 h after exercise. There were no main effects of time or treatment for: cAMP response element-binding protein, extracellular signal-regulated kinases 1/2, c-Jun N-terminal kinases, nuclear factor kappa-light-chain-enhancer of activated B cells, p38 mitogen-activated protein kinases, ribosomal protein S6 kinase beta-1, and signal transducer and activator of transcription 3 and 5 (Table 2), demonstrating that these phosphoprotein signaling pathways were not induced by the exercise.

## 4. Discussion

To our knowledge, this is the first study to examine the effects of PEA supplementation on recovery from muscle damaging exercise. In contrast to our hypothesis, we observed that PEA supplementation immediately before and after exercise did not reduce pain and localized swelling through a reduction in pro-inflammatory intramuscular enzymes and cytokines; although there was a significant reduction in blood lactate and myoglobin concentrations following PEA supplementation. PEA supplementation also increased protein kinase B phosphorylation immediately post exercise. 

### 4.1. Leg Press Exercise

The leg press exercise increased blood lactate concentration in both the PEA and placebo groups, peaking at 7.38 ± 3.09 and 8.81 ± 2.44 mmol/L, respectively. This demonstrated that the exercise was at a high intensity and increased glycolytic metabolism [16], which is similar to other reported values [17].

The exercise bout also induced muscle damage, evidenced by the increased subjective rating of muscle soreness, thigh circumference, and biomarkers (myoglobin and creatine kinase), which are all commonly found to increase after muscle damaging exercise [1,2]. The inflammatory biomarker tumor necrosis factor-α also increased following exercise, which is similar to some [18], but not all [19,20] previous studies that investigated resistance-based exercise.

There was no change in the other biomarkers of muscle damage (lactate dehydrogenase) or inflammation (high sensitivity C-reactive protein and interleukin-10 and 6) following the leg press exercise. The responses of lactate dehydrogenase [21,22], C-reactive protein [23], and interleukin-10 [24] to resistance exercise are variable. Interleukin-6 usually increases after resistance exercise, but it may be that the volume of the leg press exercise was too little to see any changes in this biomarker [23]. Lastly, other than protein kinase B phosphorylation, there were no changes in any other phosphoprotein signaling pathways measured after exercise. This may be that the stress of the exercise was not great enough to induce these signal transduction pathways [25,26].

### 4.2. Effects of PEA Supplementation

We hypothesized that PEA supplementation immediately before and after exercise would reduce pain and localized swelling through a reduction in pro-inflammatory intramuscular enzymes and cytokines. Our hypothesis was based on evidence from several human studies that investigated PEA as a treatment for pain and a recent systematic review and meta-analysis, that identified 10 studies including data from 786 participants who received PEA and 512 controls, demonstrated that PEA supplementation was associated with significantly greater pain reduction compared to inactive control conditions [9]. The mechanisms responsible for the reduction in pain may be due to PEA agonism of peroxisome proliferator activated receptor-α, which was shown to have a major role in the PEA mechanisms for pain relief [10]. PEA also has an essential role in the suppression of inflammation by reducing the activity of the pro-inflammatory enzyme’s cyclooxygenase and endothelial and inducible nitric oxide synthases [11], and by reducing mast cell activation [12,13]. The mechanisms responsible for this anti-inflammatory effect were investigated more recently. PEA inhibits phosphorylation of kinases involved in activation of pro-inflammatory pathways, such as c-Jun N-terminal kinase and extracellular signal-regulated kinases, and the nuclear translocation of the transcription factors kappa-light-chain-enhancer of activated B cells [27,28]. However, we did not see a reduction in markers of inflammation or evidence of the upregulation in phosphoprotein signaling pathways. The reason we did not observe a reduction in inflammation may be due to several reasons. Firstly, our supplementation protocol was acute and not chronic as undertaken in previous studies [12,13,29]. Secondly, we measured systemic rather than localized inflammation impacting the resolution of inflammation measured. Finally, the volume of exercise may have been insufficient to elevate all inflammatory markers, and therefore any intervention would be unable to reduce these.

PEA was provided in an acute context to investigate its short-term effects on muscle recovery. As inflammation is needed for long-term muscle growth and PEA supposedly acts in an anti-inflammatory mechanism, then chronic administration would possibly provide a negative stimulus for chronic exercise adaptation.

We observed myoglobin to be lower for the PEA group (*p* < 0.05; Figure 5) compared to the placebo. Myoglobin is one of the most commonly used markers of skeletal muscle damage and represents a proxy marker of damage to the muscle cell membrane [30]. When muscle is damaged, as with resistance exercise, myoglobin leaks into the circulation due to muscle cell disruption and, therefore, circulating concentrations of myoglobin are frequently used as markers of exercise-induced muscle damage [30]. There are several possible explanations for the discovery that PEA may reduce myoglobin concentration. Firstly, the reduction in myoglobin concentration might represent a decrease in muscle damage resulting from modification of cytokine synthesis in response to PEA. However, we think this is unlikely since, there were no changes to the cytokines measured after PEA supplementation compared to the placebo. Secondly, PEA may reduce muscle protein breakdown, possibly due to an enhanced insulin response and muscle protein turnover. Finally, another possible explanation is that PEA supplementation promotes the clearance of myoglobin from circulation.

There are conflicting results in previous research on the relationship between muscle damaging exercise, myoglobin, and other markers of muscle damage. One study by Isaacs et al. describes that only “high responders”, those whose creatine kinase (CK)increased above 1000 U/L after muscle damaging exercise, had a corresponding increase in Mb. It was also noted that only “high responders” also increased C-reactive protein (CRP) [31]. However, those that were “low responders” did not have a corresponding increase in CK or CRP suggesting that changes in Mb, CK, and CRP have a non-linear relationship. This could be why we observed a reduction in Mb but no changes in CK or CRP. We are unsure as to the mechanism between PEA and the reduction in Mb, but no congruent reduction in other markers of muscle damage was found.

Peak blood lactate concentration was lower following PEA supplementation compared to the placebo. A lower blood lactate concentration correlates with increased aerobic energy metabolism and decreased anaerobic energy metabolism. This could be due to either a decreased lactate net production or an increased net lactate uptake into surrounding tissues. It is unknown what the mechanism is for reduced lactate concentration post-exercise in the PEA group. Supplementation with PEA might therefore allow exercise to be maintained at a higher intensity for longer. It could be suggested that consumption of PEA may allow for higher exercise intensities to be achieved after an initial exercise session. This would allow for an improved training response or sports performance, particularly for those who exercise or compete within quick succession (within a few hours).

## 5. Conclusions

To our knowledge, this is the first study to examine the effects of PEA supplementation on recovery from muscle-damaging exercise. In contrast to our hypothesis, we observed that PEA supplementation immediately before and after leg press exercise did not reduce pain and localized swelling through a reduction in pro-inflammatory intramuscular enzymes and cytokines. This may be because of the volume of the exercise and/or the acute supplementation protocol used. PEA was however able to reduce myoglobin and increase protein kinase B phosphorylation following exercise. Taken together, these results may indicate that PEA supplementation is able to aid in muscle recovery from repeat bouts of exercise performed within 3 h.

## Figures and Tables

**Table 1:** Baseline participant characteristics for palmitoylethanolamide (PEA) and placebo groups. Values are mean ± SD.

	PEA	Placebo
Age (years)	27 ± 4	26 ± 4
Height (m)	1.79 ± 0.10	1.80 ± 0.10
Weight (kg)	84 ± 12	83 ± 15
Body mass index (kg/m^2^)	25 ± 2.6	26 ± 2.8
Systolic blood pressure (mmHg)	123 ± 9	124 ± 10
Diastolic blood pressure (mmHg)	76 ± 4	75 ± 11
Waist circumference (cm)	87 ± 7	87 ± 9
Hip circumference (cm)	102 ± 7	102 ± 7
Waist to hip ratio	0.86 ± 0.04	0.85 ± 0.05

**Table 2:** Phosphoprotein signaling pathways for PEA and placebo groups. Values are mean ± SD, # *p* < 0.05 compared with pre-exercise.

		Pre-exercise	Post-exercise	+1 h	+2 h	+3 h	+24 h	+48 h	+72 h
PKB (MFI)	PEA	4682 ± 2933	9362 ± 4185^#^	6146 ± 4147	5355 ± 3694	5972 ± 3313	6263 ± 4015	6163 ± 3667	4461 ± 3772
	Placebo	4444 ± 1749	4996 ± 4642	5405 ± 2574	4911 ± 2625	4798 ± 2634	4832 ± 2515	4494 ± 2053	5298 ± 2226
CREB (MFI)	PEA	98.5 ± 43.7	88.4 ± 61.7	92.7 ± 63.3	87.3 ± 51.0	122 ± 123	115 ± 105	73.0 ± 28.1	165 ± 222
	Placebo	66.4 ± 29.7	58.3 ± 27.5	94.8 ± 99.6	65.7 ± 20.7	74.5 ± 24.6	68.4 ± 38.3	62.1 ± 22.1	66.8 ± 29.6
ERK 1/2 (MFI)	PEA	39.6 ± 13.7	37.9 ± 15.8	40.3 ± 14.0	43.6 ± 13.7	40.9 ± 12.2	43.3 ± 15.0	40.2 ± 16.7	42.9 ± 13.0
	Placebo	38.2 ± 12.1	41.5 ± 13.2	44.5 ± 14.6	44.0 ± 13.8	42.0 ± 12.1	40.5 ± 12.7	41.9 ± 14.7	38.9 ± 14.9
JNK (MFI)	PEA	115 ± 92	106 ± 102	135 ± 101	120 ± 140	162 ± 133	105 ± 120	117 ± 119	120 ± 97
	Placebo	103 ± 98	78 ± 64	138 ± 114	107 ± 93	126 ± 107	101 ± 93	87 ± 70	99 ± 93
NF-κB (MFI)	PEA	17.9 ± 4.1	21.6 ± 8.1	17.8 ± 5.6	19.5 ± 9.1	21.0 ± 11.4	23.9 ± 17.9	17.1 ± 4.5	19.0 ± 7.2
	Placebo	16.4 ± 5.7	18.0 ± 5.6	20.4 ± 7.0	17.4 ± 4.1	20.3 ± 7.1	19.0 ± 4.9	20.9 ±4.1	22.3 ± 8.5
p38MAPK (MFI)	PEA	6188 ± 1791	6762 ± 1245	6184 ± 1545	5605 ± 2759	6879 ± 1706	6790 ± 2830	5772 ± 2896	5283 ± 2705
	Placebo	5926 ± 2047	6080 ± 1933	6415 ± 1698	6504 ± 1923	6556 ± 1455	6448 ± 2629	6782 ± 2061	5643 ± 2524
RPS6KB1 (MFI)	PEA	68.3 ± 70.9	47.5 ± 26.6	46.5 ± 22.6	49.6 ± 34.1	51.0 ± 31.3	42.6 ± 12.0	49.4 ± 36.4	55.5 ± 23.3
	Placebo	39.7 ± 13.0	30.3 ± 11.8	45.0 ± 29.0	37.7 ± 10.2	38.1 ± 11.1	32.9 ± 15.2	46.0 ± 42.9	44.1 ± 17.5
STAT3 (MFI)	PEA	51.9 ± 26.8	49.7 ± 19.8	55.6 ± 26.5	48.9 ± 22.7	48.6 ± 19.3	51.1 ± 19.3	43.4 ± 15.5	52.4 ± 27.0
	Placebo	42.5 ± 18.3	42.0 ± 16.2	44.0 ± 24.6	46.2 ± 24.0	48.4 ± 28.9	45.5 ± 26.5	43.2 ± 26.0	47.4 ± 19.9
STAT5 (MFI)	PEA	47.2 ± 32.4	46.3 ± 18.9	48.8 ± 20.7	50.8 ± 30.5	53.4 ± 25.9	42.8 ± 14.6	45.6 ± 22.8	41.1 ± 11.9
	Placebo	38.5 ±10.1	40.4 ± 12.4	37.7 ± 6.5	39.3 ± 13.4	39.9 ±9.9	38.3 ± 6.5	40.6 ± 9.4	38.9 ± 12.3

Abbreviations: MFI, mean fluorescence intensity; PKB, protein kinase B; CREB, cAMP response element-binding protein; ERK 1/2, extracellular signal–regulated kinases 1/2; JNK, c-Jun N-terminal kinases; NF-κB, nuclear factor kappa-light-chain-enhancer of activated B cells; p38MAPK, p38 mitogen-activated protein kinases; RPS6KB1, ribosomal protein S6 kinase beta-1; STAT, signal transducer and activator of transcription.

**Table 3:** Thigh circumference and biomarkers of muscle damage and inflammation for palmitoylethanolamide (PEA) and placebo groups. Values are mean ± SD.

		Pre-exercise	Post-exercise	+1 h	+2 h	+3 h	+24 h	+48 h	+72 h
Thigh Circumference (cm)	PEA	57.3 ± 3.93	58.5 ± 3.72	57.6 ± 3.78	57.6 ± 3.69	57.7 ± 3.71	57.5 ± 3.83	56.9 ± 3.82	57.5 ± 3.81
	Placebo	56.2 ± 4.81	57.4 ± 5.08	56.4 ± 4.60	56.3 ± 4.78	56.5 ± 4.92	56.5 ± 4.51	56.7 ± 4.40	56.6 ± 4.85
Creatine Kinase (IU/L)	PEA	213 ± 119	228 ± 130	256 ± 223	257 ± 213	261 ± 210	310 ± 220	298 ± 233	233 ± 225
	Placebo	180 ± 91	204 ± 106	196 ± 101	204 ± 97	232 ± 107	261 ± 100	226 ± 88	211 ± 138
Lactate Dehydrogenase (IU/L)	PEA	217 ± 99	183 ± 42	193 ± 44	204 ± 48	176 ± 54	164 ± 55	170 ± 27	197 ± 51
	Placebo	182 ± 30	211 ± 44	184 ± 61	202 ± 86	201 ± 84	162 ± 24	178 ± 31	173 ± 22
HS C-reactive Protein (mg/L)	PEA	1.52 ± 2.12	1.60 ± 2.16	1.39 ± 1.88	1.49 ± 2.07	1.36 ± 1.83	1.24 ± 1.49	1.41 ± 1.49	1.09 ± 1.45
	Placebo	1.26 ± 0.85	1.28 ± 0.87	1.17 ± 0.81	1.14 ± 0.79	1.15 ± 0.78	1.17 ± 1.04	1.17 ± 0.84	1.20 ± 0.95
Interleukin-10 (pg/mL)	PEA	12.1 ± 10.1	20.1 ± 31.6	17.0 ± 17.2	15.9 ± 17.4	15.9 ± 16.8	13.5 ± 10.7	14.4 ± 12.0	12.2 ± 11.6
	Placebo	16.8 ± 22.0	17.5 ± 24.0	16.0 ± 21.8	15.4 ± 17.2	14.4 ± 17.6	11.6 ± 11.1	12.5 ± 11.6	12.6 ± 10.0
Interleukin-6 (pg/mL)	PEA	12.0 ± 9.43	12.2 ± 9.12	10.4 ± 7.23	11.6 ± 8.09	11.7 ± 7.56	11.4 ± 7.17	10.8 ± 7.01	10.6 ± 7.52
	Placebo	6.23 ± 4.93	6.59 ± 4.29	5.13 ± 3.69	5.44 ± 3.71	5.92 ± 3.79	5.54 ± 3.86	5.50 ± 3.73	5.20 ± 3.38
Tumor necrosis factor-α (pg/mL)	PEA	8.03 ± 5.51	9.17 ± 7.22	7.25 ± 4.48	7.69 ± 4.61	7.45 ± 3.40	8.38 ± 4.13	7.16 ± 3.53	7.41 ± 3.51
	Placebo	8.58 ± 3.72	9.36 ± 3.11	7.80 ± 2.41	7.76 ± 3.50	7.97 ± 3.14	8.07 ± 2.57	7.52 ± 1.88	7.75 ± 2.34
